# A clinical study of Yiqi Huayu Jiedu decoction reducing the risk of postoperative gastric cancer recurrence and metastasis: Study protocol for a randomized controlled trail

**DOI:** 10.1097/MD.0000000000021775

**Published:** 2020-08-14

**Authors:** Yue Hu, Xiaoting Pan, Mengjun Nie, Yuanjie Liu, Xi Zou, Shenlin Liu, Qin Liu, Ruiping Wang, Li Zhang

**Affiliations:** a The Affiliated Hospital of Nanjing University of Chinese Medicine, Jiangsu Province Hospital of Chinese Medicine; b No.1 Clinical Medical College, Nanjing University of Chinese Medicine, Nanjing, Jiangsu, China; c Nanjing Drum Tower Hospital.

**Keywords:** postoperative gastric cancer, randomized controlled trail, traditional Chinese medicine, yiqi huayu jiedu decoction

## Abstract

**Background:**

Gastric cancer is a common gastrointestinal tumor, seriously threatening human health. Radical surgery is the preferred treatment for gastric cancer. However, due to the late diagnosis and postoperative recurrence and metastasis, the prognosis is dismal. In China, traditional Chinese medicine (TCM) has been used to treat gastric cancer for many years. The purpose of this study is to explore the efficacy and safety of Yiqi Huayu Jiedu decoction in the treatment of postoperative gastric caner.

**Methods/design:**

226 eligibility patients altogether will be randomly allocated to the treatment group and the control group at a ratio of 1:1. After enrollment, every patients will obtain 6 months of treatment, as well as 2 years of follow-up. At the end of this study, primary outcomes including 1-year progression-free survival rate, 2-year progression-free survival rate and disease-free survival, secondary outcomes containing tumor markers, TCM syndrome points, quality of life scale, imageological examination and the safety indicators will be assessed.

**Discussion:**

This study will provide the evidence-based evidence for the efficacy of Yiqi Huayu Jiedu decoction reducing the risk of postoperative gastric cancer recurrence and metastasis, which will be beneficial to form the therapeutic regimen in postoperative gastric cancer with integrated TCM and Western medicine.

**Trail registration:**

ChiCTR2000032802

## 1 Introduction

Although the incidence of gastric cancer has been declining annually in recent years, it is still 1 of the most common malignant tumors worldwide. According to statistics, there were over 1,000,000 new cases and 783,000 deaths in 2018, making it the huge obstacle for improving life expectancy.^[1]^ Geographical differences occur in the incidence and mortality of gastric cancer (GC), the highest incidence rates are in East Asia, East Europe and part of South America, especially in China.^[2]^ It is estimated that there are approximately 400,000 new cases each year in China, accounting for about 40% of new cases globally.^[3,4]^ The current major therapeutic measures of GC are surgical resection, chemotherapy, radiotherapy and biologic therapeutic agents. Due to the lack of radical treatment for advanced GC, early diagnosis and surgery is still crucial to treat it. However, even after complete resection and appropriate adjuvant therapy, postoperative recurrence and metastasis are still as high as 50% to 70%,^[5,6]^ and the survival rate of recurrent GC is fairly low.^[7,8]^

Traditional Chinese medicine (TCM), as 1 of the methods of comprehensive cancer therapies, has been widely used in clinical. It is generally accepted that TCM has its unique role in reducing the therapy-related side effects,^[9]^ prolonging the survival of cancer patients^[10]^ and improving the quality of life for cancer patients.^[11]^ The literature to date illustrates that the mechanism of TCM in the treatment of malignant tumors mainly includes immunomodulation,^[12]^ inhibition of angiogenesis,^[13]^ alteration of inflammation response^[14]^ and modification of microenvironment.^[15]^ Therefore, we hypothesized that for postoperative GC patients, TCM may reduce the risk of recurrence and distant metastasis.

Yiqi Huayu Jiedu decoction (YQHYJD) is a formula proposed by Professor Shenlin Liu, the famous Chinese physician of TCM. It is created based on the classic prescription of TCM, Gui Shao Liu Jun Zi decoction, and Prof. Liu's clinical experience. As is shown in Table 1, 13 commonly used Chinese herbal medicine comprise the YQHYJD. And our previous studies revealed that YQHYJD appears to benefit GC patients, it has a certain effect on improving symptoms, like stomachache, anorexia, and quality of life. However, whether YQHYJD can reduce the risk of postoperative GC recurrence and metastasis has not been verified. Therefore, it is necessary to explore the anti-tumor effects and safety of YQHYJD systematically.

**Table 1:**
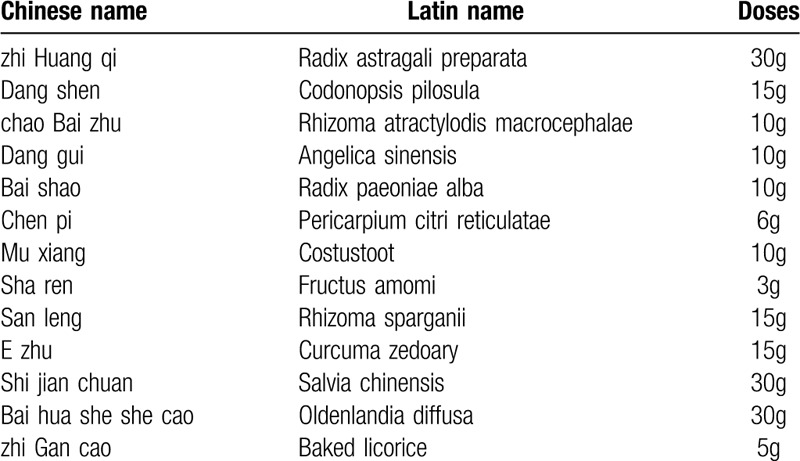
“Yiqi Huayu Jiedu decoction”(YQHYJD) composition.

## 2 Methods/design

### 2.1 Study design

**Figure 1:**
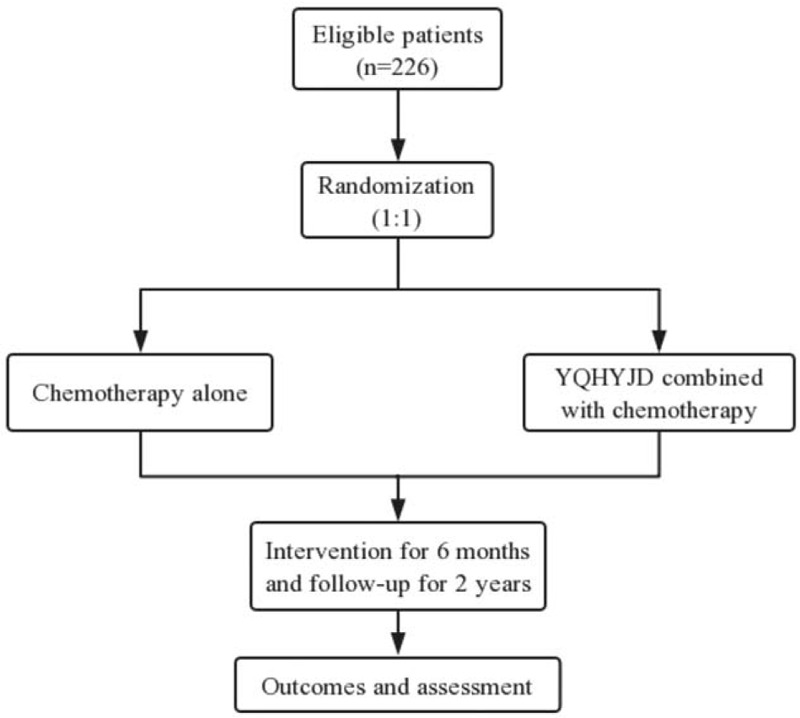
The study flowchart. The flowchart of enrollment, interventions and analysis.

This is a prospective, double-center, randomized controlled clinical trail to investigate the efficacy and safety of YQHYJD on postoperative GC recurrence and metastasis. It has been registered in Chinese Clinical Trail Registry (www.chictr.org.cn ChiCTR2000032802) on May 11, 2020. A total of 226 eligibility patients in 2 locations, including Affiliated Hospital of Nanjing University of Chinese Medicine and Nanjing Drum Tower Hospital, will be randomly enrolled in this trail at a ratio of 1:1, 113 in the treatment group (YQHYJD combined with chemotherapy) and 113 in the control group (chemotherapy alone). All the patients will receive 6 months of treatments and be followed up for 2 years. The design of this clinical trail is in accordance with the standard protocol items: Recommendation for Interventional Trails (SPIRIT) 2013 and the flowchart of this trail is shown in Figure 1. The specific evaluations and visits will be conducted according to the schedule of the study process listed in Table 2.

**Table 2:**
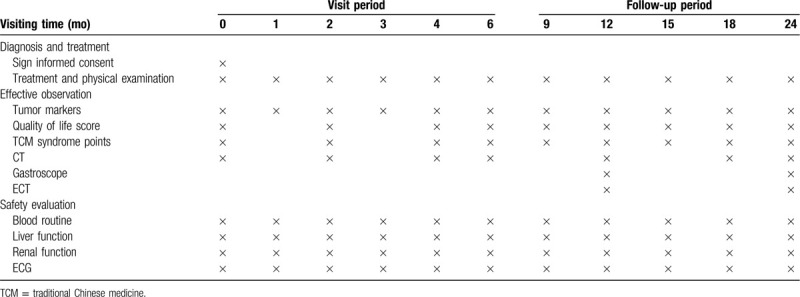
Study schedule.

This study protocol is in compliance with the Declaration of Helsinki (Edinburgh 2000 version) strictly. So far, we have achieved the approval from the Ethic Committee of Affiliated Hospital of Nanjing University of Chinese Medicine (Number 2020NL-021–03).

### 2.2 Recruitment and consent

Between July 2020 and December 2020, a total of 226 postoperative GC patients who match the screening criteria will be recruited in this clinical trail. The purpose, procedures, possible risks, benefits and other information about this trail will be explained to the participants in detail. The written consent will be obtained from each eligibility participants before enrolment and the names or the identifying information about the participants will not be revealed. In addition, all the participants have the right to withdraw from the trail at any time without any consequence.

### 2.3 Randomization

Stratified blocked randomization will be used to grouping. With the help of SAS statistical software, the randomization sequence will be generated. Then all the patients will be enrolled in the treatment group or the control group at a ratio of 1:1 according to the predetermined random number.

### 2.4 Inclusion criteria and Exclusion criteria

#### 2.4.1 Inclusion criteria

(1) Patients with pathological diagnosis confirmed GC.
(2) Patients whose pathological stage are II or III after resection.
(3) Patients who are Qi deficiency of spleen according to the TCM syndrome differentiation.
(4) Karnofsky score > 60.
(5) Patients aged from 18 to 70-years-old.
(6) Patients with an expected survival time more than 6 months.
(7) Patients who are voluntary to participate in this study and provide written informed consent according to the GCP criteria.

#### 2.4.2 Exclusion criteria

(1) Patients with metastatic GC.
(2) Patients with local recurrence or distant metastasis identified by histopathology or imaging examination.
(3) Patients who are pregnant or nursing.
(4) Patients with severe primary cardiovascular disease, liver disease, kidney disease, blood disease or other diseases influencing survival.
(5) Patients with disabilities, including the blind, the deaf, dysgnosia, mental disturbance and extremity disability.
(6) Patients who are alcohol dependence or substance abuse.
(7) Patients with situations that reduce the possibility of enrollment or complicate the enrollment, such as frequent changes in working environment.
(8) Patients who are allergic constitution,such as those who are allergic to more than 2 drugs and food, or those who are allergic to the ingredients of YQHYJD.
(9) Patients who are participating in the other clinical trails.

### 2.5 Interventions

#### 2.5.1 The control group

After radical gastrectomy, patients in the control group will be only administered with chemotherapy. The chemotherapy regimens are in accordance with NCCN guidelines and CSCO guidelines as follows (any 1 of them, 4 courses and above):

XELOX: Oxaliplatin 130 mg/m^2^ iv, on day1

Capecitabine 1000 mg/m ^2^ po, on days1-14, twice daily, every 3weeks

SOX: Oxaliplatin 130 mg/m^2^ iv, on day1

S-1 40 mg/m^2^ po, on days1-14, twice daily, every 3weeks

DS-1: Docetaxel 40-50 mg/m^2^ iv, on day1, day15

S-1 40-60 mg/m^2^ po, on days1-14, twice daily, every 4weeks

FLOFOX: Oxaliplatin 85 mg/m^2^ iv, on day1

Calcium folinate 400 mg/m^2^ iv, on day1

FU 400 mg/m^2^ iv, on day1, then 2400-3600 mg/m^2^/d civ46 h, every 3weeks.

#### 2.5.2 The treatment group

Patients in the treatment group will be treated with YQHYJD decoction as well as chemotherapy. Patients will take YQHYJD decoction at least for 6 months, 2 times a day, 200 mL each time, while the chemotherapy regimens are as same as the control group. Also, symptomatic treatment and nutrition support therapy is available when necessary.

### 2.6 Outcome measures

#### 2.6.1 Primary outcomes

The primary outcomes of this study are 1-year progression-free survival rate, 2-year progression-free survival rate and disease-free survival(DFS). All of these indicators will be caculated at the end of the trail.

#### 2.6.2 Secondary outcomes

Tumor markers: tumor markers like CEA, CA199, colsely related to the treatment of GC, will be examined before the treatment, once a month during treatment and every 3 months until the 18th month during the follow-up period. After that it will be detected every 6 months until patients’ disease progressed.

TCM syndrome points: in order to observe the curative effect of YQHYJD on symptoms, the changes of TCM syndrome will be assessed with questionnaires. The questionnaire includes 7 common symptoms of GC: stomachache, gaseous distention, low food intake, belching, sour regurgitation, eating obstruction and fatigue. Each of them has 4 levels, the normal, the mild, the moderate and the severe, corresponding to the points of 0, 3, 6, 9. The higher the point, the more severe the symptom.

Quality of life scale: the quality of life will be measured with EORTC Core Quality of Life Questionnaire (EORTC QLQ-C30), a questionnaire composed of 5 functional subscales, 3 symptom subscales, a general health status subscale and several single items. In functional and general health subscales, the higher the score, the better the quality of life, while in the symptom subscales, it is the opposite.

Imageological examination: chest and abdomen CT, gastroscopy and emission computed tomography (ECT) will be used to evaluate whether the disease progressed.

#### 2.6.3 Safety assessments

Blood routine, liver function (ALT, AST), renal function (BUN, Scr) and ECG will be used regularly to monitor the safety of YQHYJD decoction. During the clinical trail and the follow-up period, any adverse events related to this trail occurred should be documented in “Adverse events form” in detail. The researchers will decide the treatment and whether to terminate the observation or quit the clinical trail depending on the severity of patients’ condition.

### 2.7 Sample size calculation

The sample size is calculated based on the past clinical experience and the primary endpoints. According to our previous study,^[16]^ the disease-free survival rate of postoperative GC patients in the control group (chemotherapy alone) is 50.42%, while in the treatment group it is 64.14%. The sample size is calculated using the following formula: 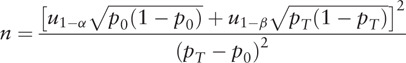


Considering that the minimum error clinical accepted is 3.5%, set α to 0.05 and the number of patients is required to achieve 80% power. Assuming a withdraw rate of 20%, allocating patients at a ratio of 1:1, the sample size of this study is eventually determined to be 226.

### 2.8 Statistical analysis

Statistician who are unrelated to this study will be responsible for the statistical analysis and the experimental data will be analyzed by SAS 9.13. For qualitative data like sex, chi-square test will be adopted. While for quantitative data, if it conforms to normal distribution, then t test will be performed, otherwise it will have measured by Wilcoxon rank sum test. Moreover, Kaplan-Meier curve will be used to compare the survival time and survival rate between the 2 groups. All statistical tests will be 2-sided test and *P*≤ .05 will be statistically significant, *P* ≤.01 will be considered highly statistically significant.

### 2.9 Data management

Information related to this study will be recorded in the CRFs in detail by a trained and qualified researcher. Once the CRF completed, it will be reviewed and signed by the main research of their unit. After that, it will be rechecked by the project sponsor within 1 week. Data will be imported into the database by the specific person and the paper versions of all the CRFs will be stored away in archives. In addition, 2 independent inspectors are responsible for checking the consistency of the source document and electronic CRF data. To ensure the authenticity of the data, the database will be locked when data entry and review are finished.

## 3 Discussion

GC is a life-threatening disease, which not only seriously affects patients’ quality of life, but also increases social burdens. TCM plays an important role in cancer therapy in China for many years, because of its anti-cancer effect and low probability of adverse effects. The essence of TCM lies in treatment based on the differentiation of disease syndromes. This RCT study is aimed to assess the efficacy of YQHYJD on postoperative recurrence and metastasis by comparing the outcomes between the YQHYJD plus chemotherapy and chemotherapy alone group. In this basis, a clinical plan for the treatment of GC with integrated TCM and Western medicine will be formed. Meanwhile, this study still has some limitations. For example, only 1 region (Nanjing) involves in this study. Whether our findings apply to other areas remain unclear. In conclusion, we expect that this study will provide a higher level of evidence-based evidence for TCM treatment of GC, which will be beneficial to lay the foundation for the development of GC treatment guidelines with Chinese characteristics.

## Author contributions

**Conceptualization**: Yue Hu

**Funding acquisition**: Li Zhang

**Formal analysis**: Mengjun Nie, Yuanjie Liu

**Investigation**: Xi Zou, Qin Liu, Yue Hu, Li Zhang

**Project administration**: Ruiping Wang

**Visualization**: Xiaoting Pan

**Supervision**: Shenlin Liu

**Writing – original draft**: Xiaoting Pan

**Writing – review & editing**: Yue Hu
